# Emergence of Azithromycin Resistance Mediated by Phosphotransferase-Encoding *mph*(A) in Diarrheagenic Vibrio fluvialis

**DOI:** 10.1128/mSphere.00215-19

**Published:** 2019-06-12

**Authors:** Goutam Chowdhury, Thandavarayan Ramamurthy, Amit Ghosh, Shanta Dutta, Eizo Takahashi, Asish K. Mukhopadhyay

**Affiliations:** a Department of Bacteriology, ICMR-National Institute of Cholera and Enteric Diseases, Kolkata, India; b Translational Health Science and Technology Institute, Faridabad, India; c Collaborative Research Center of Okayama University for Infectious Diseases at ICMR-NICED, Kolkata, India; JMI Laboratories

**Keywords:** azithromycin resistance, diarrhea, pulsed-field gel electrophoresis, *Vibrio fluvialis*, multidrug resistance

## Abstract

The azithromycin resistance conferred by phosphotransferase is encoded in the gene *mph*(A). This gene has been discovered in and reported for many bacterial species. We examined the prevalence of azithromycin resistance in Vibrio fluvialis (AR-VF) isolated during 2014 to 2015 from the hospitalized acute diarrheal patients in Kolkata, India. Most of the V. fluvialis isolates are identified as the sole pathogen (54%). The prevalence of AR-VF was higher in 2015 (19 [68%]) than in 2014 (9 [32%]). Among AR-VF isolates, the azithromycin MICs ranged from 4 to >256 mg/liter. Twenty-eight of the 48 (58%) V. fluvialis isolates harbored the gene *mph*(A) and phenotypically resistant to azithromycin. All the AR-VF isolates remained susceptible to doxycycline. In addition to azithromycin, other antimicrobial resistance-encoding genes of AR-VF were also characterized. All the AR-VF isolates were positive for class 1 integron, and most of them (17/28) carried the *dfrA1* gene cassettes. Only one isolate was positive for the *ereA* gene, which encodes resistance to erythomycin. The majority of the isolates were resistant to β-lactam antibiotics (*bla*_OXA-1_ [96%], *bla*_OXA-7_ [93%], and *bla*_TEM-9_ [68%]) and aminoglycoside actetyltransferase, conferring resistance to ciprofloxacin-modifying enzyme [*aac(6′)Ib-cr*] (96%). Analyses by pulsed-field gel electrophoresis (PFGE) showed that the AR-VF isolates belonged to different genetic lineages. This is the first study to report azithromycin resistance and the presence of the *mph*(A) gene in V. fluvialis isolates. Circulation of AR-VF isolates with high azithromycin MICs is worrisome, since it may limit the treatment options for diarrheal infections.

**IMPORTANCE** The progressive rise in antibiotic resistance among enteric pathogens in developing countries is becoming a big concern. India is one of the largest consumers of antibiotics, and their use is not well regulated. V. fluvialis is increasingly recognized as an emerging diarrheal pathogen of public health importance. Here we report the emergence of azithromycin resistance in V. fluvialis isolates from diarrheal patients in Kolkata, India. Azithromycin has been widely used in the treatment of various infections, both in children and in adults. Resistance to azithromycin is encoded in the gene *mph*(A). Emerging azithromycin resistance in V. fluvialis is a major public health challenge, and future studies should be focused on identifying ways to prevent the dissemination of this antibiotic resistance gene.

## Abstract (precis)

The progressive rise in antibiotic resistance among enteric pathogens in developing countries is becoming a big concern. India is one of the largest consumers of antibiotics, and their use is not well regulated. V. fluvialis is increasingly recognized as an emerging diarrheal pathogen of public health importance. Here we report the emergence of azithromycin resistance in V. fluvialis isolates from diarrheal patients in Kolkata, India. Azithromycin has been widely used in the treatment of various infections, both in children and in adults. Resistance to azithromycin is encoded in the gene *mph*(A). Emerging azithromycin resistance in V. fluvialis is a major public health challenge, and future studies should be focused on identifying ways to prevent the dissemination of this antibiotic resistance gene.

## INTRODUCTION

Vibrio fluvialis is a halophilic Gram-negative bacterium commonly found in coastal waters and seafood. In recent years, V. fluvialis has been recognized to cause cholera-like diarrhea. Several outbreaks and sporadic cases of acute diarrhea caused by this pathogen have been reported worldwide (1–4). The molecular characterization of this pathogen has given several insights, including its clonality and multidrug resistance (MDR) features (2, 4). The emergence and spread of antibiotic resistance represent one of the most important public health concerns and are correlated with the inappropriate use of antimicrobial agents, which results in increased mortality, morbidity, and health care costs (5). Antibiotic resistance arises through several modes, such as transfer of plasmids, integrons, and transposons, mutations in target genes, and the overexpression of efflux systems (5). The rapid increase and spread of antibiotic resistance in V. fluvialis during the past 20 years are a major cause of concern because of the organism’s ability to cause epidemics (6).

We have reported the existence of extended-spectrum β-lactamases (ESBLs) and fluoroquinolone-acetylating aminoglycoside-6′-*N*-acetyltransferase in V. fluvialis, which confers the resistance to fluoroquinolones and β-lactam antimicrobials (7), including *bla*_NDM-1_-mediated carbapenem resistance (8). Here we report the identification and characterization of an azithromycin resistance (AR) macrolide 2 P-phosphotransferase I-encoding gene, *mph*(A), in 28 isolates of V. fluvialis (AR-VF) isolated during 2014 to 2015 from hospitalized patients in Kolkata, India. Azithromycin belongs to the macrolide class of antibiotics, which are primarily used to treat infections caused by Gram-positive microorganisms. This antibiotic is active also against several Gram-negative organisms (9). Considering the emergence of MDR enteric bacteria, azithromycin is recommended for the treatment of diarrhea (10, 11). Isolates with higher MICs of azithromycin were found to harbor *mph*(A) (12). From the previous studies, it is known that the *mph*(A) gene has been disseminated among different pathogens, like Escherichia coli, *Salmonella* spp., and *Shigella* spp. (13, 14). The present study was undertaken to understand the azithromycin resistance mechanisms in V. fluvialis isolates from diarrheal patients in Kolkata.

## RESULTS

During 2014 to 2015, a total of 48 V. fluvialis were isolated from 2,308 (1,135 and 1,173 received in 2014 and 2015, respectively) stool specimens of acute diarrheal patients, of which 28 (58%) were found to be resistant to azithromycin. The isolation rates of AR-VF were 19% in 2014 (9 of 48 isolates) and 39% (19 of 48 isolates) in 2015. Patients from whom V. fluvialis was isolated presented cholera-like diarrhea, i.e., with watery stool (67%), severe dehydration (14%), and abdominal pain (50%) (Table 1). Out of the 28 isolates of AR-VF (each strain representing a case), 15 (54%) were isolated as the sole pathogen and the remaining 13 (46%) were recovered along with other pathogens, such as Vibrio cholerae, diarrheagenic Escherichia coli (DEC), *Shigella* spp., *Salmonella*, *Campylobacter* spp., parasites, and viruses. Among the polymicrobial etiology cases, V. fluvialis along with V. cholerae O1 were found to be present at a relatively high proportion (14%), followed by DEC and *Shigella* spp. (11% each). AR-VF infection was more often detected in adults (82%) than in children <5 years of age (18%). The other enteric pathogens isolated along with AR-VF remained susceptible to azithromycin (data not shown).

**TABLE 1:** Clinical features of patients with diarrhea who were infected with azithromycin-resistant V. fluvialis

Clinical features	No. (%) of patients
Pathogen	
V. fluvialis as sole infecting organism	15 (53.6)
Mixed infection with V. fluvialis and other organisms	13 (46.4)
V. cholerae O1	4 (14.2)
*Shigella* spp.	3 (10.7)
DEC	3 (10.7)
Campylobacter jejuni	1 (3.5)
Rotavirus	1 (3.5)
Giardia lamblia	1 (3.5)
Age	
>5 yrs	23 (82.1)
≤5 yrs	5 (17.9)
Sex	
Male	17 (60.8)
Female	11 (39.2)
Type of diarrhea	
Watery	19 (67.9)
Bloody mucus and loose	9 (32.1)
Dehydration status	
Severe	4 (14.3)
Some and rare	24 (85.7)
Abdominal pain	
Yes	14 (50)
No	14 (50)

The results of antimicrobial susceptibility testing and MIC values for AR-VF isolates are presented in Table 2. The majority of the AR-VF isolates were also resistant to ampicillin (100%), erythromycin, cefotaxime (CTX), ceftriazone, and sulfamethoxazole (96% each), nalidixic acid and streptomycin (93% each), norfloxacin, ciprofloxacin, and ofloxacin (89% each), gentamicin (68%), ceftadizime (CAZ; 61%), chloramphenicol (36%), meropenem (18%), and tetracycline (14%). It was also seen that the AR-VF displayed a wide range of MIC values for most of the antibiotics (Table 2).

**TABLE 2:** Antimicrobial resistance profiles, integrons, and MICs of AR-VF isolates^*a*^

Strain	Resistance	Class 1 integron ORF size (kb)	Antibiotic resistance gene(s)	MIC (μg/ml)									
				E	AZM	CRO	CIP	NOR	GM	SXT	MEM	NA	TE
IDH 06261	AM, AZM, CRO, E, GM, CTX, NA, S, SXT, CIP*, OFX*, N*, NOR*	1.2	*dfrA1*, *orfC*	64	96	>256	12	24	24	>32	ND	>256	ND
IDH 06263	AM, AZM, CIP, CRO, E, GM, CTX, OFX, NOR, NA, S, SXT, N*	2.5	*arr2*, *cm1A5*, *aac(6′)-Ib*	64	96	>256	12	24	24	>32	ND	>256	ND
IDH 06279	AM, AZM, CIP, CRO, E, GM, CTX, OFX, NOR, NA, S, SXT, N*	2.5	*arr2*, *cm1A5*, *aac(6′)-Ib*	64	128	>256	32	16	16	>32	ND	>256	ND
IDH 06350	AM, AZM, CIP, CRO, C, E, CAZ, CTX, OFX, NOR, NA, S, SXT, N*	1	*aadA13*	>256	>256	>256	8	16	ND	>32	ND	>256	ND
IDH 06430	AM, AZM, E, GM, CTX, SXT, CRO, S*	1.5	*vfsbp*	256	256	>256	ND	ND	64	>32	ND	ND	ND
IDH 06639	AM, AZM, CIP, CRO, E, CAZ, GM, CTX, OFX, NOR, NA, S, SXT	1.2	*dfrA1*, *orfC*	192	>256	>256	32	24	48	>32	ND	>256	ND
IDH 06736	AM, AZM, CIP, CRO, C, E, CAZ, CTX, OFX, NOR, NA, S, SXT, N*	1.2	*dfrA1*, *orfC*	48	128	>256	32	24	ND	>32	ND	>256	ND
IDH 06848	AM, CIP, CRO, E, CAZ, GM, CTX, MEM, OFX, NOR, NA, S, SXT, AZM*	1.2	*dfrA1*, *orfC*	12	4	>256	>32	64	16	>32	32	>256	ND
IDH 06915	AM, AZM, CIP, CRO, E, CAZ, GM, CTX, MEM, OFX, NOR, NA, S, SXT	1.2	*dfrA1*, *orfC*	>256	48	>256	>32	256	16	>32	>32	>256	ND
IDH 07410	AM, E, GM, CTX, S, SXT, AZM*, CRO*	1.5	*vfsbp*	>256	4	256	ND	ND	24	>32	ND	ND	ND
IDH 07715	AM, AZM, CIP, CRO, C, E, CAZ, GM, CTX, OFX, NOR, NA, S, SXT	1.2	*dfrA1*, *orfC*	128	256	>256	8	16	48	>32	ND	>256	ND
IDH 07930	AM, AZM, CIP, CRO, C, E, CAZ, GM, CTX, MEM, OFX, NOR, NA, S, SXT	1.5	*vfsbp*	>256	4	3	1	48	0.75	>32	0.75	>256	ND
IDH 07931	AM, AZM, CIP, CRO, C,E, CAZ, GM, CTX, MEM, OFX, NOR, NA, S, SXT	0.8	*aac(6′)-Ib*	>256	32	>256	>32	24	8	>32	3	>256	ND
IDH 07968	AM, AZM, CIP, CRO, C, E, GM, CTX, OFX, NOR, NA, S, SXT	1.2	*dfrA1*, *orfC*	128	256	>256	32	16	16	>32	ND	>256	ND
IDH 07978	AM, CIP, CRO, C, E, CAZ, CTX, OFX, NOR, NA, S, SXT, AZM*, GM*	1.5	*vfsbp*	>256	48	3	1	3	0.75	>32	ND	>256	ND
IDH 07986	AM, CIP, CRO, C, E, CAZ, CTX, OFX, NOR, NA, S, SXT, GM*, AZM*	1.5	*vfsbp*	>256	4	3	3	3	0.75	>32	ND	>256	ND
IDH 07992	AM, AZM, CIP, CRO, E, CAZ, CTX, OFX, NOR, NA, S, SXT, GM*	1.2	*dfrA1*, *orfC*	256	>256	>256	>32	64	8	>32	ND	>256	ND
IDH 08037	AM, AZM, CIP, CRO, E, CAZ, GM, CTX, OFX, NOR, NA, S, SXT, C*, MEM*, N*, TE*	1.2	*dfrA1*, *orfC*	128	256	>256	>32	48	64	>32	8	>256	8
IDH 08125	AM, AZM, CIP, CRO, C, E, CAZ, GM, CTX, OFX, NOR, NA, S, SXT, MEM*, N*, TE*	1.2	*dfrA1*, *orfC*	128	96	>256	>32	32	48	>32	8	>256	6
IDH 08126	AM, AZM, CIP, CRO, E, CAZ, GM, CTX, OFX, NOR, NA, S, SXT, C*, TE*	1.2	*dfrA1*, *orfC*	128	128	>256	>32	32	32	>32	ND	>256	6
IDH 08128	AM, AZM, CIP, CRO, E, CAZ, GM, CTX, OFX, NOR, NA, TE, S, SXT, C*, MEM*, N*	1.2	*dfrA1*, *orfC*	96	256	>266	>32	48	>256	>32	16	>256	8
IDH 08164	AM, AZM, CIP, CRO, C, E, CAZ, CTX, MEM, OFX, NOR, NA, S, SXT, N*	1.2	*dfrA1*, *orfC*	128	>256	>256	12	24	6	>32	4	>256	ND
IDH 08180	AM, AZM, CIP, CRO, E, CAZ, GM, CTX, OFX, NOR, NA, S, SXT, MEM*, N*	1.2	*dfrA1*, *orfC*	>256	256	>256	>32	64	6	>32	4	>256	ND
IDH 08181	AM, CIP, CRO, E, CAZ, CTX, OFX, NOR, NA, S, SXT, AZM*	1.5	*vfsbp*	96	4	32	>32	48	ND	0.5	ND	>256	ND
IDH 08223	AM, AZM, CIP, CRO, E, CAZ, GM, CTX, OFX, NOR, NA, S, SXT, N*	1.2	*dfrA1*, *orfC*	>256	12	>256	3	4	2	1.5	0.75	>256	ND
IDH 08273	AM, CIP, CRO, E, CAZ, GM, CTX, OFX, NOR, NA, S, SXT, AZM*, MEM*, N*	0.8	*aac(6′)-Ib*	>256	12	>256	12	24	32	>32	ND	>256	ND
IDH 08299	AM, CIP, CRO, E, CTX, OFX, NOR, NA, S, C*, SXT*, AZM*	1.2	*dfrA1*, *orfC*	16	0.5	>256	>32	32	ND	>32	ND	>256	ND
IDH 08349	AM, AZM, CIP, CRO, E, CAZ, CTX, OFX, NOR, NA, SXT, C*, MEM*, N*	1.2	*dfrA1*, *orfC*	>256	48	>256	>32	32	ND	>32	6	>256	ND

a ORF, open reading frame; MEM, meropenem; CTX, cefotaxime; CIP, ciprofloxacin; NOR, norfloxacin; TE, tetracycline; AM, ampicillin; E, erythromycin; SXT, trimethoprim-sulfamethoxazole; NA, nalidixic acid; S, streptomycin; C, chloramphenicol; GM, gentamicin; OFX, ofloxacin; CRO, ceftriaxone; AZM, azithromycin; CAZ, ceftazidime; N, neomycin; ND, MIC assay was not done for isolates susceptible to respective antibiotics. Asterisks indicate drugs to which the isolate showed reduced susceptibility.

Five distinct gene cassettes were identified in the class 1 integron-bearing AR-VF isolates, which include (i) 2 isolates with a 2.5-kb amplicon comprising *arr2* (rifampin resistance), *cm1A5* (chloramphenicol resistance), and *aac(6′)Ib-cr* (ciprofloxacin resistance); (ii) 6 isolates with 1.5-kb gene cassettes with the *vfsbp* gene, which confers resistance to class 1 integron periplasmic pectic oligomer binding protein; (iii) 17 isolates with 1.2-kb gene cassettes containing *dfrA1* (trimethoprim resistance) and *orfC*; (iv) 1 isolate with a 1-kb product with the presence of *aadA13*, encoding aminoglycoside-3′-adenylyltransferase; (v) 2 isolates with a 0.8-kb product with *aac(6′)Ib*, which codes for aminoglycoside *6′-N*-actetyltransferase.

Overall, 8 different resistance gene profiles were identified (Table 3). All the AR-VF isolates harbored *mph*(A). The resistance gene *ere*(A) was detected in one isolate (IDH 08223), and all remaining AR-VF isolates were negative for *mph*(B), *erm*(A), *erm*(B), *erm*(C), *ere*(A), *ere*(B), *mef*(A), and *msr*(A). Most of the AR-VF isolates also harbored *bla*_OXA-1_ (96%), *bla*_OXA-7_ (93%), and *bla*_TEM-9_ (68%), conferring resistance to β-lactamase, as well as *aadB* (96%; gentamicin resistance), *aac(6′)Ib-cr* (96%; ciprofloxacin resistance), and *aadA1* (96%; streptomycin resistance)*. bla*_CTX-M-3_, which confers resistance to ceftriaxone, was detected in 19 (68%) AR-VF isolates. The streptomycin and sulfonamide resistance-encoding genes *strA* and *sul2* were detected in 24 (86%) and 22 (79%) isolates, respectively. The *bla*_NDM-1_ gene, which encodes resistance to carbapenem, was found in 11 (39%) isolates, and macrolide-modifying enzymes, such as esterase encoded by *ere*(A), were detected in an isolate.

**TABLE 3:** Distribution of *mph*(A) gene along with other antimicrobial resistance genes in AR-VF isolates

Strain	Presence of integron and macrolide and other resistance genes												
	*int1*	*mph*(A)	*aac(6´)-Ib-cr*	*bla_Oxa-1_*	*aadB*	*aadA1*	*bla*_OXA-7_	*bla*_CTX-M-3_	*bla*_TEM-9_	*strA*	*sul2*	*bla*_NDM-1_	*ere*(A)
IDH 07930	+	+	+	+	+	+	+	+	+	+	+	+	−
IDH 07931	+	+	+	+	+	+	+	+	+	+	+	+	−
IDH 08037	+	+	+	+	+	+	+	+	+	+	+	+	−
IDH 08125	+	+	+	+	+	+	+	+	+	+	+	+	−
IDH 08128	+	+	+	+	+	+	+	+	+	+	+	+	−
IDH 08273	+	+	+	+	+	+	+	+	+	+	+	+	−
IDH 06263	+	+	+	+	+	+	+	+	+	+	+	−	−
IDH 06279	+	+	+	+	+	+	+	+	+	+	+	−	−
IDH 06736	+	+	+	+	+	+	+	+	+	+	+	−	−
IDH 07978	+	+	+	+	+	+	+	+	+	+	+	−	−
IDH 07986	+	+	+	+	+	+	+	+	+	+	+	−	−
IDH 08126	+	+	+	+	+	+	+	+	+	+	+	−	−
IDH 08299	+	+	+	+	+	+	+	+	+	+	+	−	−
IDH 07715	+	+	+	+	+	+	+	+	+	+	+	−	−
IDH 07968	+	+	+	+	+	+	+	+	+	+	+	−	−
IDH 07992	+	+	+	+	+	+	+	+	+	+	+	−	−
IDH 06261	+	+	+	+	+	+	+	+	+	+	+	−	−
IDH 06430	+	+	+	+	+	+	+	−	−	+	+	−	−
IDH 06639	+	+	+	+	+	+	+	−	−	+	+	−	−
IDH 07410	+	+	+	+	+	+	+	−	−	+	+	−	−
IDH 08181	+	+	+	+	+	+	+	−	−	+	+	−	−
IDH 06848	+	+	+	+	+	+	+	+	+	−	−	+	−
IDH 06915	+	+	+	+	+	+	+	+	+	−	−	+	−
IDH 08164	+	+	+	+	+	+	+	−	−	+	−	+	−
IDH 08180	+	+	+	+	+	+	+	−	−	+	−	+	−
IDH 08349	+	+	+	+	+	−	+	−	−	−	+	+	−
IDH 08223	+	+	+	+	+	+	−	−	−	−	−	−	+
IDH 06350	+	+	−	−	−	+	−	−	−	+	−	−	−

Pulsed-field gel electrophoresis (PFGE) was performed to investigate the genetic relationship between AR-VF isolates. Twenty-four different PFGE patterns were detected (data not shown). Distinct lineages were identified between the isolates and MDR patterns, suggesting that the AR-VF isolates were genetically diverse.

## DISCUSSION

Globally, multidrug resistance in enteric bacterial pathogens is an emerging threat to public health (5). Infection caused by V. fluvialis has been increasingly reported worldwide (6), with frequent documentation of MDR (15, 16). The prevalence of MDR in V. fluvialis organisms varies depending on the country and the source of isolation. A study from South Africa reported the prevalence of V. fluvialis resistant to ampicillin, penicillin G, streptomycin, sulfamethoxazole, trimethoprim, chloramphenicol, erythromycin, ciproﬂoxacin, and polymyxin B (1). The presence of a relatively high number of V. fluvialis isolates that were resistant to β-lactams and sulfamethoxazole has been reported from China (2). Previously, we have shown the presence of a transmissible MDR plasmid in V. fluvialis isolates possessing several antimicrobial resistance-encoding genes, i.e., *aac(3)-IIa*, *bla*_NDM-_*_1_*, *bla*_TEM-1_, *dfrA15*, *sul1*, *tet*(A), *floR*, *strAB*, and *sul2* (8, 16). In this study, we identified azithromycin-resistant V. fluvialis with high MIC values. AR-VF emerged in Kolkata at a low level during late 2013 and subsequently increased during 2014 to 2015 (32 to 67%) among diarrheal patients. This is the first report of the emergence of azithromycin resistance in V. fluvialis.

Azithromycin has been recommended for the treatment of acute diarrhea caused by *Campylobacter* spp., DEC, *Shigella* spp., nontyphoidal *Salmonella*, and sometimes V. cholerae (17). Due to the wide use of this antibiotic, resistance or reduced susceptibility to azithromycin has increased in several enteric pathogens, such as Escherichia coli, *Salmonella* spp., and *Shigella* spp. (12, 18, 19).

The AR-VF isolates were characterized using phenotypic and molecular techniques to understand the mechanism of resistance and their clonal relationships. Several studies have already reported that macrolide resistance has been acquired through a variety of mechanisms. In many Gram-negative bacteria, macrolide resistance is primarily involved with target site modification by methylases encoded by *erm* [*erm*(A), *erm*(B), and *erm*(C)] genes, macrolides inactivated by modifying esterase enzyme [*ere*(A) or *ere*(B)] genes, phosphotransferases encoded by *mph* [*mph*(A) and *mph*(B)] genes, and acquisition of efflux pump [*mef*(A) and *msr*(A)] genes. The association of these genes with complete cross-resistance of erythromycin and azithromycin has been reported frequently (12, 13, 20). Considering this, we have targeted these nine genes for macrolide resistance while screening the V. fluvialis isolates that showed resistance to azithromycin. We detected *mph*(A) in 28 isolates and *ere*(A) in 1 isolate; no other macrolide resistance-encoding genes were detected. Twenty-eight of 48 isolates examined in this study harbored the *mph*(A) gene and had azithromycin MICs between 4 and >256 mg/liter. One isolate that had both the *ere*(A) and *mph*(A) genes showed an azithromycin MIC of >256 mg/liter. These results indicate that carriage of *mph*(A) alone can confer higher azithromycin resistance in V. fluvialis. This is similar to previous observations made in *Shigella* spp. and *Salmonella* spp. (13, 14).

All the AR-VF isolates possessed class 1 integrons, of which 17 had *dfrA1* and *orfC*. The presence of the *dfrA1* gene cassette, coding for trimethoprim resistance in V. fluvialis, has been reported previously (7, 15). As reported before (16), the *vfspb* gene, which encodes periplasmic pectic oligomer binding protein (Sbp), was found in the class 1 integrons of six AR-VF isolates. A 2.5-kb amplicon obtained from 2 AR-VF isolates (IDH 06263 and IDH 06279) showed the presence of *arr2* and *cm1A5*, coding for rifampin and chloramphenicol resistance. Interestingly, the presence of these genes has been reported for the integrons of Acinetobacter baumannii isolates from East Africa (21). The major ESBL genes, like *bla*_OXA-1_ (96%), *bla*_OXA-7_ (93%), bla_TEM-9_ (68%), and bla_CTX-M-3_ (68%), were identified in most of the AR-VF isolates. This observation further suggests that the major ESBL groups are prevalent in V. fluvialis in Kolkata (7). Since these ESBL genes are generally located on antimicrobial resistance plasmids, they can easily be disseminated between different species of bacteria (22). The other enteric pathogens isolated along with AR-VF were susceptible to azithromycin. This indicates the genomic suitability of V. fluvialis in the quick acquisition of the *mph*(A) gene.

In this study, we have shown that most of the AR-VF organsims isolated in Kolkata harbor various antimicrobial resistance-encoding genes. AR-VF isolates belonged to different lineages, indicating their diverse sources of infection. This is a cause of major concern, as multidrug resistance could pose a challenge both for the treatment and prevention of spread of infections caused by V. fluvialis. It is important to monitor the genes responsible for the resistance to azithromycin and other antimicrobials in V. fluvialis and other enteric bacteria isolated as copathogens.

## MATERIALS AND METHODS

### Bacterial isolates.

Twenty-eight azithromycin-resistant V. fluvialis isolated from patients with cholera-like diarrhea admitted to Infections Diseases, Kolkata, between 2014 and 2015 were included in this study. V. fluvialis was isolated from stool specimens or rectal swabs in Cary-Blair medium using thiosulfate-citrate-bile salts-sucrose agar (TCBS), followed by overnight incubation at 37°C. Yellow colonies on the TCBS plates were examined by oxidase test, string test using 0.5% sodium deoxycholate solution, biochemical testing (ID 32GN; bioMérieux, Marcy l’Etoile, France) and by a species-specific V. fluvialis *toxR* PCR (4).

### Antimicrobial susceptibility testing.

Antibiotic susceptibility testing was done using the Kirby-Bauer method according to the Clinical and Laboratory Standards Institute (23). Antibiotic discs (BD, USA) used were meropenem (10 μg), ceftazidime (CAZ; 30 μg), cefotaxime (CTX; 30 μg), cefepime (30 μg), ampicillin (10 μg), azithromycin (15 μg), ceftriaxone (30 μg), chloramphenicol (30 μg), ciprofloxacin (5 μg), co-trimoxazole (25 μg), erythromycin (15 μg), gentamicin (10 μg), kanamycin (30 μg), neomycin (30 μg), tetracycline (30 μg), nalidixic acid (30 μg), norfloxacin (10 μg), ofloxacin (5 μg), and streptomycin (300 μg). Each isolate was inoculated in Mueller-Hinton broth (Difco) and incubated at 37°C for 3 h. The turbidity of the suspension was adjusted to 0.5 McFarland standard, and the suspension was then spread on a Mueller-Hinton agar (Difco) plate using a sterile swab. The plates were incubated at 37°C overnight after placement of the antimicrobial susceptibility test discs. The zone of inhibition was measured and interpreted as per the CLSI guidelines (23). Extended-spectrum-β-lactamase (ESBL) production was confirmed by double-disc synergy testing with CAZ and CTX. An increase of 5 mm in the zone diameter for CAZ or CTX compared to that for clavulanic acid was considered for production of ESBL. The MICs of meropenem, ciprofloxacin, norfloxacin, ceftazidime, cefotaxime, and cefepime were determined using Etest (AB Biodisk, Solana, Sweden), following the manufacturer’s instructions. Escherichia coli ATCC 25922 and Klebsiella pneumoniae ATCC 700603 were used as quality control strains.

### PCR.

Total DNA of the isolates was extracted using QIAamp genomic DNA kits (Qiagen, Valencia, CA), as per the manufacturer’s instructions. PCR was used to amplify the macrolide resistance genes (12). To detect other antimicrobial resistance-encoding genes for carbapenemase (*bla*_NDM-1_), gentamicin (*aadB*), streptomycin (*aadA1* and *strA*), kanamycin (*aphA1-1a*), chloramphenicol (*catA1*), β-lactams (*bla*_TEM_, *bla*_OXA-1_, *bla*_OXA-7_, *bla*_OXA-9_, *bla*_SHV_, *bla*_PSE-4_, and *bla*_CTXM-3_), ciprofloxacin [*aac(6′)-1b-cr*], and chloramphenicol (*floR*), PCR was carried out as described previously (8, 24). For the amplification of putative antibiotic resistance gene cassettes present in the class 1 integron-bearing strains, primers inf-F (5′-CS) and in-B (3′-CS) were used. All the assays were carried out in the simplex PCR format. PCR amplicons were electrophoresed on 1.5% agarose gels.

### Sequencing.

Amplified PCR products were extracted from the gel after electrophoresis and were purified using a QIAquick gel extraction kit (Qiagen) as per the manufacturer’s instructions. Purified DNA was analyzed on a 1.5% agarose gel. Sequencing reactions were performed using a BigDye Terminator v.3.1 cycle sequencing kit (Applied Biosystems, Foster City, CA). The dye terminators from sequencing reactions were removed using a DyeEx 2.0 spin kit (Qiagen). Purified sequences were analyzed using an ABI 3500 genetic analyzer (Applied Biosystems). The DNA sequences were edited with Lasergene software (DNASTAR, Inc., Madison, WI) and analyzed using the BLAST program of the NCBI database (https://www.ncbi.nlm.nih.gov/blast).

### PFGE.

Pulsed-field gel electrophoresis (PFGE) analysis of NotI-digested genomic DNA was performed using a CHEF-Mapper (Bio-Rad Laboratories, Hercules, CA) according to the PulseNet standardized PFGE protocol for subtyping of Vibrio cholerae (25). The PFGE image was captured using a Gel Doc XR system (Bio-Rad), and the gel image was normalized by aligning the peaks of the Salmonella enterica serovar Braenderup (H9182) size standard in each gel and analyzed using BioNumerics software v.4.0 (Applied Maths, Sint-Martens-Latem, Belgium). The fingerprint patterns were also subjected to typing based on banding similarity and dissimilarity using the Dice similarity coefficient. Cluster analysis was performed based on the single-linkage method. Finally, the results were graphically represented as dendrograms.
